# Mental Imagery Affects Subsequent Automatic Defense Responses

**DOI:** 10.3389/fpsyt.2015.00073

**Published:** 2015-06-03

**Authors:** Muriel A. Hagenaars, Rahele Mesbah, Henk Cremers

**Affiliations:** ^1^Department of Clinical Psychology, Behavioural Science Institute, Radboud University Nijmegen, Nijmegen, Netherlands; ^2^Department of Clinical Psychology, Leiden University, Leiden, Netherlands; ^3^Biological Science Division, Department of Psychiatry, University of Chicago, Chicago, IL, USA; ^4^Department of Depression, PsyQ Rijnmond, Rotterdam, Netherlands

**Keywords:** imagery, freezing, bradycardia, heart rate, rescripting, immobility, passive viewing paradigm, memory

## Abstract

Automatic defense responses promote survival and appropriate action under threat. They have also been associated with the development of threat-related psychiatric syndromes. Targeting such automatic responses during threat may be useful in populations with frequent threat exposure. Here, two experiments explored whether mental imagery as a pre-trauma manipulation could influence fear bradycardia (a core characteristic of freezing) during subsequent analog trauma (affective picture viewing). Image-based interventions have proven successful in the treatment of threat-related disorders and are easily applicable. In Experiment 1, 43 healthy participants were randomly assigned to an imagery script condition. Participants executed a passive viewing task with blocks of neutral, pleasant, and unpleasant pictures after listening to an auditory script that was either related (with a positive or a negative outcome) or unrelated to the unpleasant pictures from the passive viewing task. Heart rate was assessed during script listening and during passive viewing. Imagining negative related scripts resulted in greater bradycardia (neutral-unpleasant contrast) than imagining positive scripts, especially unrelated. This effect was replicated in Experiment 2 (*n* = 51), again in the neutral-unpleasant contrast. An extra no-script condition showed that bradycardia was not induced by the negative-related script, but rather that a positive script attenuated bradycardia. These preliminary results might indicate reduced vigilance after unrelated positive events. Future research should replicate these findings using a larger sample. Either way, the findings show that highly automatic defense behavior can be influenced by relatively simple mental imagery manipulations.

## Introduction

Like animals, humans freeze, fight, or flight under threat. Fight-flight reactions, characterized by heart rate acceleration (tachycardia), increased muscle tonus and action, are well known and well studied in general as well as in relation with emotional disorders. For example, panic is associated with uncoordinated behavior (an uncoordinated fight-flight response) during imminent and inescapable threat (1, 2). Moreover, the relevance of flight behavior in the etiology of threat-related disorders is generally acknowledged, which is reflected in the use of exposure as a major component in the treatment of anxiety disorders.

Although freezing behavior is well studied in animal literature, interest in human immobility responses has grown since the last decade only. Like fight-flight, freezing is part of the defensive system, although it is physically – and proposed functionally – different. Freezing is characterized by heart rate deceleration (bradycardia), increased muscle tonus, and immobility (3). It is associated with optimal perceptual and attentional processing and prepares for rapid action, thereby increasing survival chances (4–6).

Like other defense behaviors (see, for example, avoidance), immobility responses may also become dysfunctional. For example, in humans, retrospective self-reports of peri-trauma immobility suggest an association between peri-trauma immobility responses and the development of psychiatric symptoms, such as posttraumatic stress disorder [PTSD; e.g., Ref. (7)]. Trauma-analog experiments with healthy participants – using observational quasi-experimental designs (8) and experimental manipulation designs (9) – also found that self-reported immobility and immobility manipulations during an aversive film resulted in increased intrusive memories of the film. Moreover, retrospective reports of peri-trauma immobility were related to decreased treatment effect in PTSD patients (10). Clearly, more research is needed in this relatively under-explored area. Findings are relevant, but most studies used retrospective self-report indicators of immobility responses, thereby relying on memory, which is known to be affected by factors like forgetting, over-reporting, and erroneous attribution of symptoms. The use of objective indicators of freezing could solve some of these problems.

In the past decade, freezing responses have also been elicited in a laboratory setting using objective markers of freezing (heart rate reductions and decreases in body sway). Freezing indeed occurred when participants were viewing highly unpleasant stimuli, such as pictures of corpses and threatening animals (11, 12), angry faces (13), or unpleasant films (14). The passive viewing task is considered a good analog for post-encounter threat responses, as participants are confronted with threatening stimuli while not being able to escape the situation [albeit by instructions and social compliance (15)]. Cognitive factors may contribute to perceived uncontrollability of feeling trapped (16). As findings have been quite consistent not only across different stimuli but also across different research groups, the paradigm seems solid for provoking freezing-like responses.

In conclusion, as peri-trauma immobility may play a role in the development and treatment of emotional disorders, a next step would be to examine whether these automatic immobility responses could be influenced. Apart from providing tools for interventions, this would also enlighten some of the mechanisms associated with automatic defense responses. This study, therefore, examined whether automatic immobility responses (freezing) can be influenced in an analog setup.

Mental imagery refers to the experience of perception without concurrent sensory input (17). It is highly relevant with respect to PTSD, with vivid intrusive images as a key component. Moreover, mental imagery is used successfully in the main treatment strategies for PTSD. For example, in imaginal exposure (18), patients have to relive (i.e., vividly imagine) the traumatic event repeatedly, until anxiety responses habituate. Another strategy, imagery rescripting, also uses the trauma images as a basis for change. Here, patients have to mentally imagine the event, after which they are asked to intervene and alter the ending in a way that they would prefer [e.g., Ref. (19, 20)]. Here too, it is of great importance that the patients are depicting the unfolding of the event as vivid as possible, meaning that they have to create vivid mental images. Except for healthy effects of expressing inhibited responses the effects of imagery rescripting are considered to result from changing the meaning of the original event (21), or changing the UCS-UCR representation in memory (22). Although, clearly, this mechanism takes place after the event has happened, possibly, creating a different stage before the event will happen will also proactively change the meaning of the event.

The success of these two treatments may lay in the fact that the original event becomes very real by activating mental images, and as a consequence, basic alterations can be made in the memory of that event and its associated emotional responses. Brain areas activated during mental imagery greatly overlap with those activated during processing of real sensory information (23). Images were also more confused with reality than verbal thoughts (24). Mental imagery has been shown to be strongly related to emotional processing (25, 26). Mental imagery has even been shown to affect subjective feelings associated with a specific event (27) and influence future behavior (28–30). Indeed, mental imagery also affects physiological responses, such as heart rate and skin conductance (31, 32). It proved to promote fear potentiated startle as well (33).

In sum, mental imagery is widely used in the treatment of PTSD. It has shown to strongly elicit emotional responses including changes in heart rate. However, it has not been established whether it can also change physiological reactions to a later event. It is possible that negative imagery activates the defense motivational system, thereby changing automatic responses to subsequent threat. It may change the meaning of or set a stage for a subsequent event (34, 35). This study, therefore, examined whether manipulations of mental imagery before picture viewing could alter automatic autonomic responses during subsequent picture viewing. It was hypothesized that imagining a script that was related to unpleasant pictures that would later be shown, would affect automatic threat responses during picture viewing. We expected increased bradycardia during subsequent unpleasant picture viewing if the script had a negative outcome (negative related script; NR), but not when the script had a positive outcome (positive related script; PR). A positive unrelated (PU) script was used as an extra control condition in order to distinguish mood induction and changes in attribution as explanatory factors. That is, the PR script could activate a general positive mood or approach system, thereby hindering defense responses, such as bradycardia (i.e., similar effects in PR and PU conditions). Alternatively, the PR script could change the meaning of or set the context for the subsequent negative event (pictures; i.e., different effects in PR and PU conditions). For instance, participants in the PR condition would perceive the unpleasant pictures as negative but “controllable,” whereas those in the NR condition would perceive them as “uncontrollable” and those in the PU condition would be unprepared. Such distinct effects were found previously after administration of PU and PR post-“trauma” imagery interventions (34). Possibly, similar changes in meaning can also be achieved “proactively” by PR imagery by providing an alternative context before the actual event takes place. We measured changes in self-report anxiety and controlled for them in case of significant condition differences, in order to check whether the effects of the scripts would be the result of changes in mood or rather changes in attribution.

## Experiment 1

### Method experiment 1

#### Participants

A total of 43 students (two males) from Leiden University took part in the study. Participants received course credits or cash money for their participation. Age ranged from 18 to 26 years, with a mean age of 19.7 years (SD = 1.8). Participants were randomly assigned to the script conditions: PU (*n* = 13), PR (*n* = 13), and NR (*n* = 17). As a result of technical problems with the polar band system, heart rate assessments failed for 1 participant, leaving 16 participants in the NR condition. Written informed consent was obtained from all participants.

#### Material/Measures

##### Heart rate

Heart rate was recorded with a Polar s810 Heart rate Monitor. The polar band was placed at the height of the sternum. The signal was converted to beats per minute (BPM) and heart rate variability. The raw heart rate data were processed and checked with use of the Polar Precision Performance SW v.4. The Polar s810 proved valid to measure R–R intervals (36).

##### Subjective anxiety

Participants rated subjective anxiety/distress on a scale from 0 (not at all) to 10 (extremely).

##### Scripts

Three different scripts with a similar setups and similar phrases were created for this study. The PU script was not related to any of the pictures that were presented in the passive viewing task. It described how the participant takes a walk through a sunny town, thinking about where to have a coffee and hearing Summer-sounds. He/she sits down to have a drink, while people nod friendly. The PR and NR scripts were both related to the unpleasant pictures; these scripts describe the “story” of an injured victim of physical violence while the pictures showed injured and mutilated people resembling that victim. The PR script had a positive outcome. It described how the participant walks through town when hearing a loud scream. The participant runs toward the sound and finds a seriously injured person, while another person is running away. The participant responds quickly and immediately calls 911, staying with the victim until the parametrics arrive. The victim survives the attack because of this adequate action, and his family is very grateful. The NR script was similar to the PR script except that the outcome was negative. That is, after hearing the scream and running toward the event, the participant is scared and stiffens. When the participant realizes that the offenders have gone, he/she cannot make a 911 call because he/she forgot to bring his/her phone. The participants want to scream for help but are not able to make a sound. The injured person subsequently dies. The PU, PR, and NR scripts had a duration of 64, 77, and 60 s, respectively.

##### Script rating questionnaire

Participants rated their own script on vividness (How vivid was your script-induced imagery?), valence (How pleasant was the script?), and arousal (How arousing/exciting was the script?) from 0 (not at all) to 10 (extremely).

#### Passive Viewing Task

##### Pictures

Three sets of 20 stimuli were selected from the International Affective Picture System^1^ [IAPS (37)]. We used the same pictures as Hagenaars et al. (12), in order to facilitate the interpretation of the results in terms of defensive freezing. The neutral set comprised 20 pictures of neutral objects. The pleasant set comprised 20 pleasant pictures depicting people in action (e.g., in a roller coaster or doing sports). The unpleasant set consisted of 20 unpleasant pictures depicting physical violence and mutilation.

##### Picture presentation

Pictures were presented in blocks containing 20 pictures of the same valence that were shown without inter trial interval. The pictures within each block were randomized, and block order was counterbalanced. A 10-second black screen preceded the first block, and 8-second black screens were presented between the subsequent blocks. Stimuli were presented full screen at eye-height on a 17-inch computer screen, approximately 1 m in front of the participant. The experiment took place in a dimly -lit room. The total viewing time was 3 min and 26 s.

#### Procedure

Participants completed a list with some basic questions [name, gender, age, and payment-type (course credits or cash money)] at arrival in the lab after which the Polar Heart Rate Monitor was placed. The participants then listened to either PU, PR, or NR script. Scripts were presented in auditory form and participants wore headphones when listening to the scripts. Participants were instructed to close their eyes and listen attentively to the script and imagine the situation (read in the first person) as vividly as possible. Closing eyes diminishes other incoming visual information, so this way they could optimally focus on the auditory information. There was a 6-s delay before the script started so that the participants had time to concentrate and prepare for the mental imagery. Participants rated their script and level of subjective anxiety after the script ended, after which the passive viewing task was started. They again rated their anxiety after the passive viewing task, after which the Polar Heart Rate Monitor was taken off.

#### Analyses

Repeated measures Analyses of Variance (rm ANOVA) were done with valence (neutral, pleasant, and unpleasant pictures) as a within factor, script condition (PU, PR, and NR) as between factor, and heart rate as dependent variable. As fear bradycardia is a within-subjects relative response (i.e., a decrease in heart rate in response to unpleasant pictures relative to neutral or pleasant pictures), we calculated contrast scores for *post hoc* analyses [see also Ref. (12)]. All tests were two-tailed except for one-tailed tests for the main effects of unpleasant pictures on heart rate, because of clear the directional hypothesis (bradycardia) as well as the fact that opposite effects would not result in different actions (38). The significance level is set at α = 0.05. Given the small sample size, results with *p* < 0.10 are considered a trend.

### Results experiment 1

#### Manipulation Checks

##### Physiological measures during audio script

There were no differences between conditions in mean HR during listening to the audio script (F(2, 37) = 2.04, *p* = 0.14, η_p_^2^ = 0.10, see Table 1 for descriptives).

**Table 1:** **Heart rate during scripted imagery, script ratings and subjective anxiety in Experiment 1 (*n* = 42)**.

	Positive unrelated, *n* = 13	Positive related, *n* = 13	Negative related, *n* = 16
Heart rate during script	79.7 (3.3)	75.2 (3.0)	71.1 (2.7)
Script rating			
Vividness**	8.39 (0.26)	7.39 (0.26)	7.29 (0.23)
Valence***	9.00 (47)	5.08 (0.47)	3.53 (0.41)
Arousal	6.00 (0.54)	7.00 (0.54)	6.65 (0.48)
Anxiety increase*	1.67 (2.50)	0.08 (1.62)	0.27 (1.53)

*Anxiety increase = ratings post passive viewing minus ratings pre passive viewing*.

*p < 0.05.

**p < 0.01.

***p < 0.001

##### Script ratings

A MANOVA with vividness, valence and arousal as dependent factors revealed differences between conditions in script ratings (F(6, 78) = 7.40, *p* < 0.001, η_p_^2^ = 0.36). Most importantly, conditions differed on valence (F(2, 40) = 40.05, *p* < 0.001, η_p_^2^ = 0.67) but not on arousal (F(2, 40) = 0.87, *p* = 0.42, η_p_^2^ = 0.04). The PU script was rated as more pleasant than the PR and NR scripts (both *p’*s < 0.001), and the PR script as more pleasant than the NR script (*p* = 0.02). Conditions also differed on vividness (F(2, 40) = 5.65, *p* = 0.01, η_p_^2^ = 0.04), with the PU script being rated as most vivid (PR: *p* = 0.01; NR: *p* = 0.003) and no differences between the PR and NR conditions (*p* = 0.80).

##### Subjective anxiety

**Figure 1:**
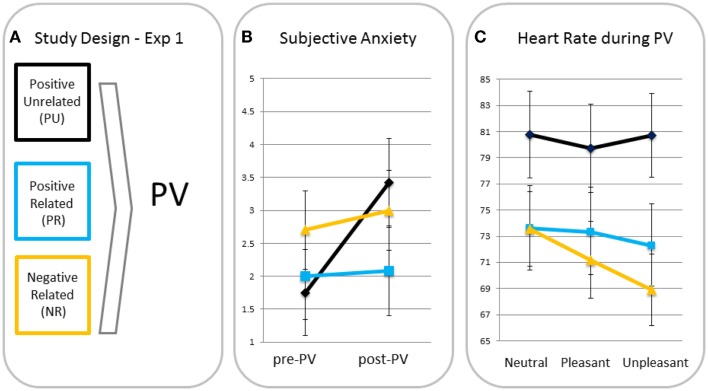
**Study design (A), subjective anxiety pre- and post-passive viewing [PV; (B)] and heart rate in response to neutral, pleasant, and unpleasant pictures (C) for participants in the positive unrelated (PU), positive related (PR), and negative related (NR) script condition**.

A rm ANOVA with time as a within factor and condition as a between factor showed a significant main effect for time (F(1, 37) = 4.19, *p* < 0.05, η_p_^2^ = 0.10), indicating anxiety increased from pre- to post-passive viewing. There was a trend for the time × condition interaction (F(2, 37) = 2.79, *p* = 0.07, η_p_^2^ = 0.13), indicating increases in anxiety tended to differ across conditions (see Figure 1). *Posthoc* analyses showed that increases in anxiety in the PU condition were greater than in the PR and NR conditions (*p* = 0.05 and *p* = 0.04, respectively). Because of these differences, pre- to post-increases in subjective anxiety were entered in the heart rate analyses as a between-subject covariate^2^. PR and NR conditions did not differ in anxiety increase (*p* = 0.96).

##### Differences in heart rate responses

A rm ANOVA showed a significant main effect of valence (F(2, 70) = 5.56, *p* = 0.006, η_p_^2^ = 0.14) with heart rate being significantly lower for unpleasant than for neutral (*p* = 0.005, one-tailed) blocks and a similar trend for the pleasant–unpleasant contrast (*p* = 0.08, one-tailed). This effect resembles the effect of Hagenaars et al. (12), who used the same task with the same pictures, suggesting a similar freezing was elicited. There was a trend for the valence × condition interaction (F(4, 70) = 2.25, *p* = 0.07, η_p_^2^ = 0.11). MANOVAs with the neutral–unpleasant and pleasant–unpleasant contrast scores as dependent variables and condition as fixed factor, showed greater heart rate decreases in the NR condition than in the PU condition in response to unpleasant versus neutral pictures (*p* = 0.02) and a similar trend for the NR relative to the PR condition (*p* = 0.06). There was no difference between PR and PU conditions (*p* = 0.49). Heart rate decreases for unpleasant versus pleasant tended to be greater for the NR relative to the PU condition (*p* = 0.08), but not relative to the PR condition (*p* = 0.50). There were no differences between the PR and PU conditions for the pleasant–unpleasant contrast (*p* = 0.28).

### Discussion experiment 1

The purpose of this study was to test whether automatic defense responses can be affected by pre-trauma mental imagery. Indeed, participants that imagined a picture-related negative scene with negative outcome experienced greater bradycardia when subsequently viewing unpleasant pictures (relative to neutral pictures) than those imagining a picture-related scene with a positive outcome, or those that imagined a PU scene. This was true for the unpleasant–neutral contrast. Moreover, there was no difference between the PR and PU groups.

The data suggest that automatic defense responses can indeed be affected by a simple and brief imagery manipulation. As both PR and PU conditions showed less bradycardia than the NR condition, it seems most likely that the two positive scripts induced a general positive mood or primed the defensive system in a positive bias rather than specifically changing the meaning of the subsequent event (unpleasant pictures). This is not in line with other findings that indicated a superior effect for trauma-related imagery with a positive outcome over non-specific positive imagery, which suggest alterations in attributions (34). However, note that participants in the NR and PU conditions tended to differ with respect to heart rate changes in the pleasant–unpleasant contrast, while NR and PR conditions did not. Speculatively, this could indicate that “attribution” processes did indeed differ for PR and PU conditions, but low power hindering the PR–PU comparison to reach significance.

In addition, both positive conditions could be effective and at the same time associated with a different mechanism. The fact that anxiety increased from pre- to post-passive viewing in the PU but not in the PR condition might suggest that this is indeed the case. Future studies should therefore replicate these findings with different tasks or measures, in order to examine whether general positive imagery (general positive mood) and specific positive imagery (changing the meaning of the event) tap into similar underlying mechanisms, for example, using memory tasks (e.g., cued recognition to assess memory accessibility) or symptom checks (e.g., intrusive images or negative cognitions about the world or self). Disentangling these working mechanisms is highly relevant, as they may have distinct short-term and long-term effects. For example, general positive imagery not affect long-term processes because it does not target the stressful event [see, for example, Ref. (34)]. In a treatment-context, this would increase relapse rates.

Another remarkable finding is that anxiety increases from pre- to post-picture viewing were greatest for the participants that listened to a PU script. One possible explanation could be that those were the participants that were least “prepared” for watching the unpleasant pictures, and thus, they felt more overwhelmed than the other groups. This may be in line with earlier results that showed that freezing was enhanced in traumatized but healthy participants (12). Our findings may also indicate that individuals become more cautious after having experienced stress. Speculatively, this may be an effective evolutionary strategy and underscores the relevance of freezing as a state of increased alertness (15). Interestingly, there were no differences in anxiety increase between the PR and NR conditions. Possibly, the relatedness may be of more relevance than the actual outcome of the script in terms of being “prepared.” Note that it is not uncommon that analog trauma does not greatly increase subjective anxiety levels [e.g., Ref. (39)], possibly indicating that the deviating result is in the PU condition.

In addition, although mental imagery had an effect on subsequent automatic responses to stress, the direction of the effect is not clear. The findings can be interpreted in two ways: (1) negative imagery elicits stronger freezing responses or (2) positive imagery reduces freezing responses. In other words, it is not clear what the “baseline response” would be. Experiment 2 was set up to address this question by replacing the PU condition by a no imagery condition (NoI).

## Experiment 2

### Method experiment 2

#### Participants

A total of 51 students from Leiden University took part in the study. Participants were randomly assigned to the script conditions and received course credits or cash money for their participation. Three participants were excluded from the analyses as being age-outliers (>3 SDs above the mean; *n* = 2) and bradycardia-outlier (>3 SDs above the mean; *n* = 1). As the heart rate signal of several participants showed many artifacts, all heart rate analyses were done by two independent persons. Participants were excluded if these analyses did not match (*n* = 6), leaving a total sample of 42 (10 males): NoI (*n* = 15), PR (*n* = 14) and NR (*n* = 13). Mean age was 23.2 years (SD = 2.4).

The design was exactly the same as in Experiment 1, with just one difference: the PU imagery manipulation was replaced by a group that received NoI (this group started the passive viewing task after completing the basic questions and placements of the electrodes). Also, heart rate was recorded continuously using the BioPack system (MP150: BIOPAC systems, Inc., CA, USA) with three matching Ag–AgCl (silver–silverchloride) electrodes. The signal was processed offline using Acknowledge software (BIOPAC systems, Inc.), and heart rate was calculated from the resulting interbeat interval (IBI).

Like in Experiment 1, all statistical tests were two-tailed except for the one-tailed testing of main effects of valence on heart rate. The significance level is again set at α = 0.05 and trend level at α = 0.10.

### Results experiment 2

#### Manipulation Checks

##### Physiological measures during audio script

Like in Experiment 1, there were no differences between conditions in mean HR during listening to the audio script (t(25) = -0.16, *p* = 0.87; see Table 2 for descriptive statistics).

**Table 2:** **Heart rate during scripted imagery, script ratings, and subjective anxiety in Experiment 2 (*n* = 42)**.

	No imagery, *n* = 15	Positive related, *n* = 14	Negative related, *n* = 13
Heart rate during script	N/A	74.5 (9.9)	75.2 (11.9)
Script rating			
Vividness	N/A	6.86 (1.83)	6.75 (1.22)
Valence*	N/A	3.50 (0.86)	2.58 (1.51)
Arousal	N/A	6.00 (1.57)	5.83 (1.80)
Anxiety increase	0.75 (1.29)	1.50 (1.51)	0.38 (2.10)

*Anxiety increase = ratings post passive viewing minus ratings pre passive viewing*.

*p < 0.1.

##### Script ratings

Like in Experiment 1, independent *t*-tests revealed no differences in vividness (*t*(24) = 0.17, *p* = 0.87) and arousal ratings (*t*(24) = 0.25, *p* = 0.80) between PR and NR conditions. There was a trend for the valence ratings to be higher in the PR than in the NR condition (*t*(24) = 1.95, *p* = 0.06).

##### Subjective anxiety

**Figure 2:**
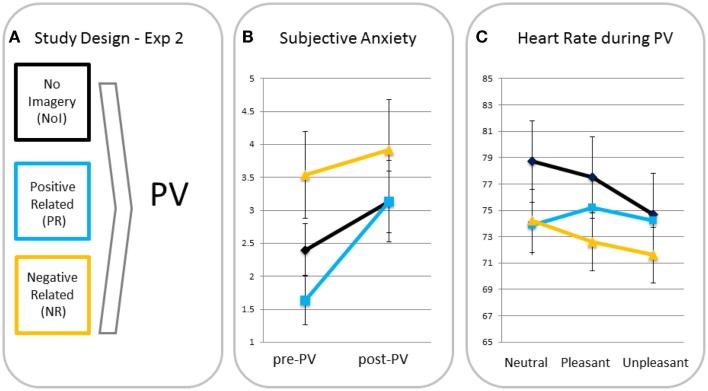
**Study design (A), subjective anxiety pre- and post-passive viewing [PV; (B)] and heart rate in response to neutral, pleasant, and unpleasant pictures (C) for participants in the no imagery (NoI), positive related (PR), and negative related (NR) script condition**.

A rm ANOVA with time as a within factor and condition as a between factor showed a significant main effect for time (F(1, 39) = 11.56, *p* = 0.002, η_p_^2^ = 0.23), indicating anxiety increased from pre- to post-passive viewing. There was no time × condition interaction (F(2, 39) = 1.61, *p* = 0.21, η_p_^2^ = 0.08), indicating there were no between-condition differences in increases in anxiety (see Figure 2). Analyses were, therefore, run without anxiety increases as a covariate^3^.

##### Differences in heart rate responses

A rm ANOVA showed a significant main effect of valence (F(2, 78) = 9.44, *p* < 0.001, η_p_^2^ = 0.20) with heart rate being significantly lower for unpleasant than for neutral (*p* < 0.001, one-tailed) and pleasant (*p* = 0.001, one-tailed) blocks. The valence × condition interaction was also significant (F(4, 78) = 3.62, *p* = 0.009, η_p_^2^ = 0.16) indicating different heart rate changes for the script conditions. MANOVAs revealed that relative to the PR condition, NR (*p* = 0.03) and NoI (*p* = 0.002) conditions showed greater heart rate decreases in response to unpleasant versus neutral pictures. There was no difference in neutral-unpleasant heart rate decreases between the NR and NoI conditions (*p* = 0.31). There were no differences between any of the conditions for the pleasant-unpleasant contrast (all *p’*s > 0.12).

### Discussion experiment 2

Experiment 2 was set up in order to replicate the findings of Experiment 1 and examine whether this effect was driven by the positive or the negative scripts. The findings of Experiment 1 were indeed replicated; for the neutral–unpleasant picture contrast, imagining an event-related script with a negative outcome (NR) induced more bradycardia than the same script with a positive outcome (PR). Moreover, the fact that bradycardia was found for the NoI condition with no differences with the NR condition indicates that this effect is driven by the positive script. The PR script seems to arrest the bradycardia response rather than the NR script increasing it.

Surprisingly, although in pilot studies as well as in Experiment 1 the NR and PR scripts differed on valence, this difference reached a trend level only in Experiment 2. One could conclude the manipulation was not successful. It is indeed remarkable that the ratings for the PR script were this low. Although the PR script describes a negative event, its ending is explicitly positive and rated as such before. There are two conclusions that have to be tested in subsequent studies: the manipulation has failed and the scripts have to be rated positive. One could examine this by comparing the participants in the PR script condition that rate the script as pleasant versus those in the PR condition that rate their script as unpleasant. Another option is that the scripts have implicit effects regardless of their explicit valence.

Note that like in Experiment 1, Experiment 2 revealed no differences in pre- to post-anxiety increases between the PR and NR conditions. Moreover, anxiety did not increase more in the NoI condition either, suggesting that indeed the “surprise-factor” induced the large anxiety increase in the PU condition in Experiment 1.

## General Discussion

Both Experiments 1 and 2 show that automatic responses to threat can be altered by mental imagery. Moreover, a simple and brief (approximately 1 min) mental imagery intervention already had an effect on subsequent autonomic responses. Especially relative to neutral pictures, participants responded with attenuated bradycardia after imagining a (non-related) positive script. Previous studies have shown that positive target-related mental imagery affected mood and future behavior (29, 30), but to our knowledge, the effects of target-related imagery on automatic autonomic responses had not been examined before.

Our findings are inconclusive about the relevance of “relatedness” of the mental imagery. Experiment 1 may suggest that mood induction – and not changes in meaning – is causing the effect, as the PU condition does not differ from the PR script condition. However, a rejection of the “relatedness”-hypothesis would be more valid if the positive conditions would not differ in valence, or if the PR condition would have been valenced more positive, which was not the case here. The fact that there was a trend for the PU condition – and not the PR condition – to differ from the NR condition in the pleasant–unpleasant contrast might suggest that relatedness is at least part of the underlying working mechanism. The difference in anxiety increase between the PU and PR condition also points in that direction. This would be in line with treatment studies that have found an additive effect of rescripting the memory over pure exposure in terms of non-fear problems (40). From a treatment perspective, the emotional processing theory posits that memory structures should be activated in order to add new information (41, 42). Possibly, forming a memory structure beforehand including event-specific knowledge may determine how the subsequent trauma information will be embedded (43). Mace (44) also suggests (involuntary) memories can be activated by cueing or “involuntary autobiographical memory chaining” (spreading of activation over related memories). This would suggest that the mental imagery script in our study would be activated automatically when watching the unpleasant IAPS pictures, automatically setting the context for these pictures. Setting a different context or altering cognitive appraisal was indeed found to affect heart rate responses to mutilation pictures as well as intrusion development and voluntary perceptual memory (35, 45, 46). However, our effects may also result from a dose response effect with the PU script being more pleasant than the PR script. Future research should disentangle whether the effects are caused by positive mood induction (impeding vigilance), by changes in meaning, for example, by using a related and non-related positive script with similar valence ratings, or including a negative unrelated-script condition. Experiment 2 cannot enlighten this issue as one positive condition was included only. Another important issue to address in future research is whether the attenuated bradycardia (i.e., less freezing) effects we found in the PU (no context) condition may to some extent explain previous memory findings (35, 47), as theoretically, automatic freezing responses have been linked to enhanced attentional and sensory processing (15).

Interestingly, one could conclude from Experiment 1 that freezing is indeed enhanced after negative imagery with the negative script condition being different from the two positive conditions, at least for the neutral–unpleasant contrast. However, the results of Experiment 2 are not in line with this conclusion. Actually, participants in the NoI condition also showed a freezing response, replicating previous findings (11, 12, 14). This is interesting because it may indicate the relevance of bradycardia or freezing in response to negative stimuli. That is, the fact that participants in the NoI condition also showed bradycardia in response to the negative pictures (Experiment 2) suggests that freezing was attenuated after an unrelated positive script rather than enhanced after the negative script. Possibly, this is the result of not being prepared for negative stimuli, as the context for these PU participants was positive and relaxed. This is in line with the idea that heart rate reductions are associated with motor preparation and enhanced attention (48) as well as increased risk assessment (49). In this context, the larger increase in anxiety for the PU script condition (Experiment 1) may also indicate a sympathetically driven response like fight/flight in the PU condition. Note that this very well matches the importance of trauma-predictability, with an unpredictable stressor resulting in greater fear and arousal than a predictable stressor (50). That is, the unpleasant pictures may have been more unpredictable for those in the unrelated-script condition, where a general positive context was induced.

Our results do not mean that decreased heart rate may be more functional. Responses in all conditions may have been adequate: Bradycardia may be functional in participants that are alert to danger (because of the imagery in the PR and NR conditions or because of the general experimental instructions warning for unpleasant pictures in the NoI condition). On the other hand, heart rate increases may be functional when one is surprised or confronted with unpredicted negative material and action is needed. Our results are in line with a previous study showing freezing was attenuated in healthy participants with no prior aversive event, whereas those with one or more aversive events showed freezing (12). Note that these were all healthy participants, and enhanced freezing may indicate preparedness and/or resilience. Interestingly, PTSD patients showed almost immediate heart rate increases in response to unpleasant pictures (51). At first sight, this may seem contradictory to the link between peri-trauma immobility and PTSD that was found in several studies [e.g., Ref. (7)]. However, immobility responses may have a different role in the prediction and maintenance of PTSD. For example, speculatively, increased immobility may predict PTSD by inducing feelings of guilt (52), whereas decreased risk assessment and increased sympathetic activation (hence decreased freezing and increased fight/flight) may act in the maintenance of PTSD. Also, different immobility responses (e.g., freezing versus tonic immobility) may play a distinct role in the etiology of PTSD (3, 14).

Clearly, results should be interpreted with caution, as both Experiments 1 and 2 suffered from power problems. Careful conclusions may be justified though, as the results point in the same direction. Future replication studies are needed with larger sample sizes. Future studies are also merited in order to enlighten the underlying working mechanisms, for example, by using a negative unrelated script or general mood inductions, or by indexing implicit and explicit memory afterwards to examine its relationship with the autonomic responses during threat. Future studies should add a measure of positive mood, which would help in distinguishing alterations in meaning versus mood effects. Finally, including symptom inventories and specific populations would also enlighten underlying mechanisms. For example, imagery rescripting was found to be effective for high anxiety individuals mostly and autonomic responses to mutilation pictures were attenuated after a safety signal for participants with high positive affect only (46, 53). However, as autonomic responses are important predictors for PTSD development, this might be a first step in implementing mental imagery in populations that will encounter traumatic events such as military, police, and the fire brigade. Note, though, that at this stage it is not clear what the most beneficial type of imagery would be: an unrelated script inducing attenuated freezing and anxiety or a related (negative) script that induces freezing. Future studies should address that issue.

In sum, interventions based on mental imagery have been proven effective in the treatment of several psychiatric disorders, for example, PTSD. Although in need of replication, our findings indicate that a brief and simple mental imagery manipulation can alter autonomic responses to subsequent threat. The findings contribute to an increased understanding of the role of mental imagery in the development of clinical disorders.

## Conflict of Interest Statement

The authors declare that the research was conducted in the absence of any commercial or financial relationships that could be construed as a potential conflict of interest.

## Footnotes

^1^Following IAPS catalog numbers for pictures are used in this study: neutral: 7000, 7002, 7004, 7006, 7009, 7010, 7020, 7025, 7030, 7031, 7035, 7040, 7050, 7052, 7060, 7080, -7090, 7150, 7175, 7211; Pleasant: 8021, 8032, 8034, 8040, 8041, 8090, 8161, 8186, 8190, 8192, 8200, 8210, 8300, 8370, 8400, 8460, 8465, 8467, 8470, 8620; Unpleasant: 3000, 3010, 3030, 3051, 3053, 3060, 3061, 3062, 3063, 3064, 3069, 3080, 3100, 3102, 3110, 3130 3140, 3150, 3261, 3400.

^2^Analyses without anxiety increases as a covariate yielded similar results.

^3^As an extra check, analyses were also done with anxiety increases as a covariate, just like in Experiment 1. Findings are similar to the ones reported here.
